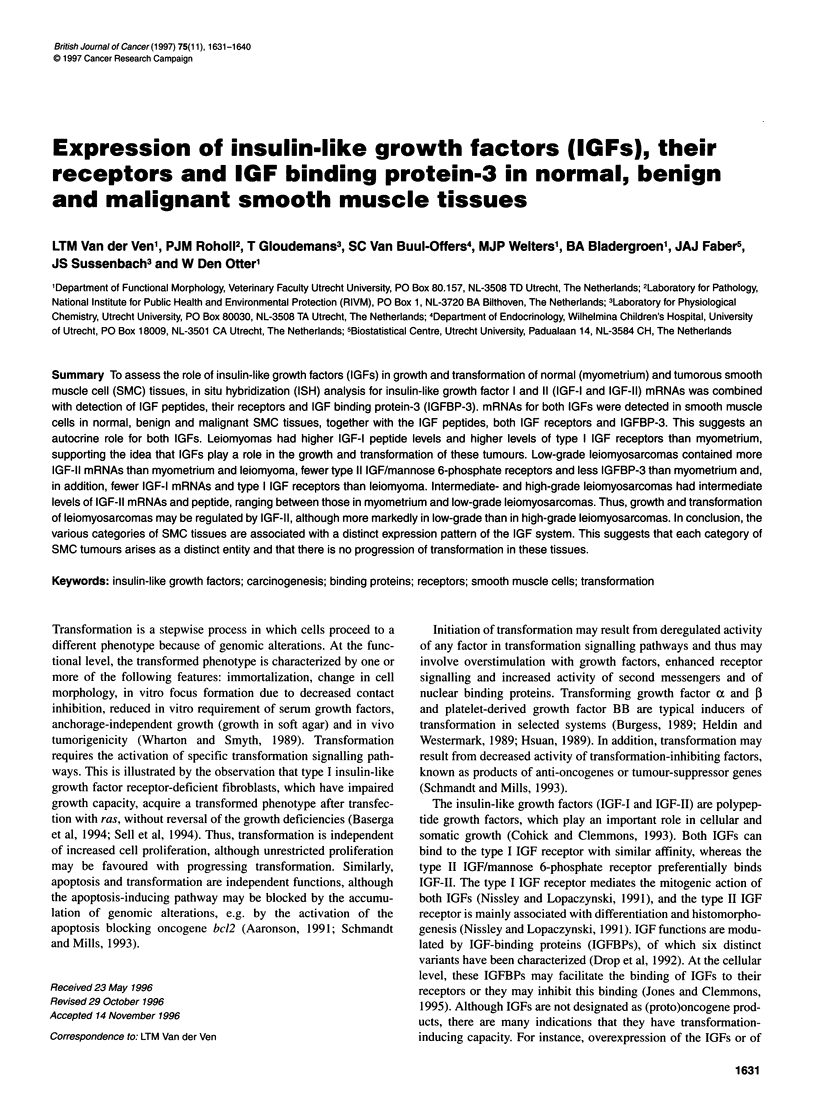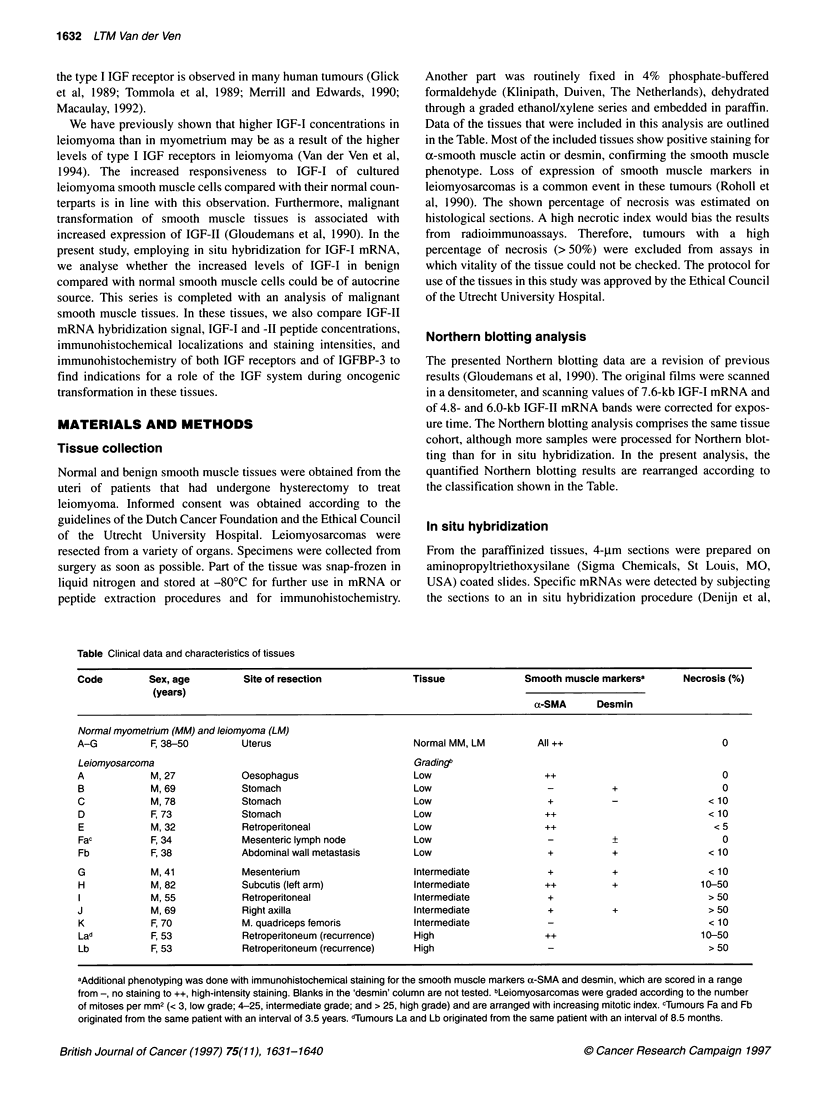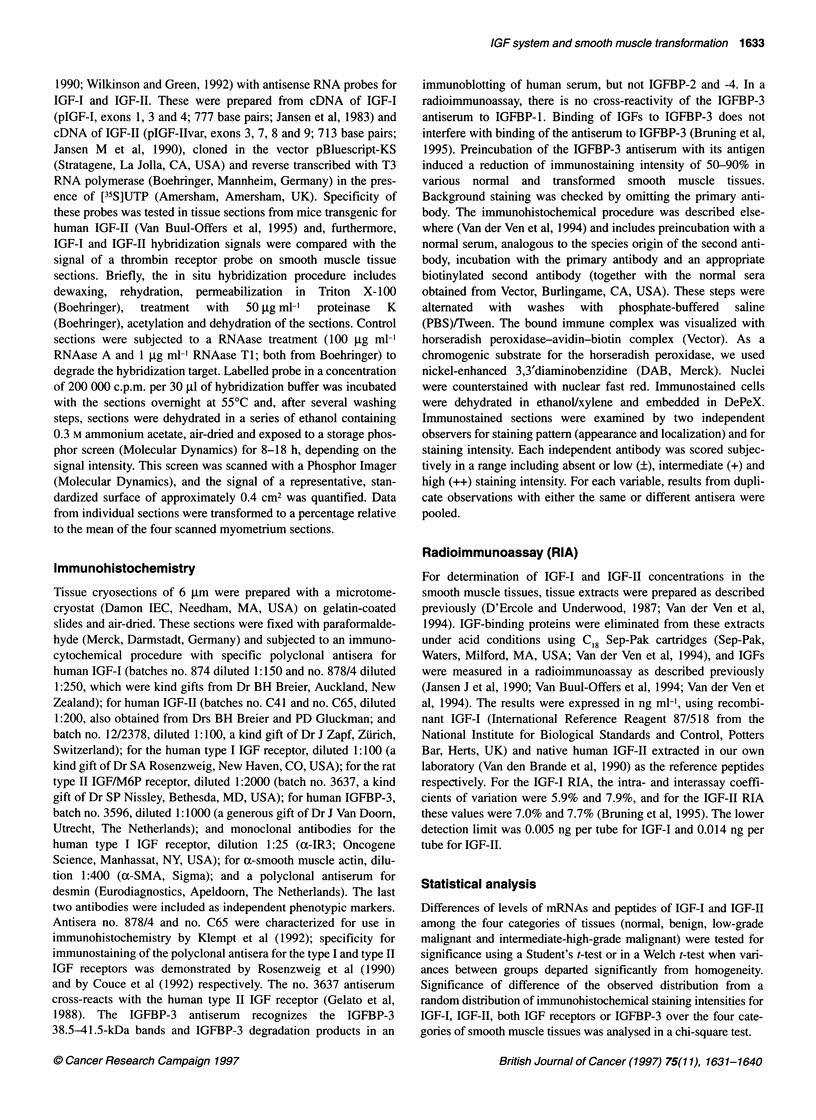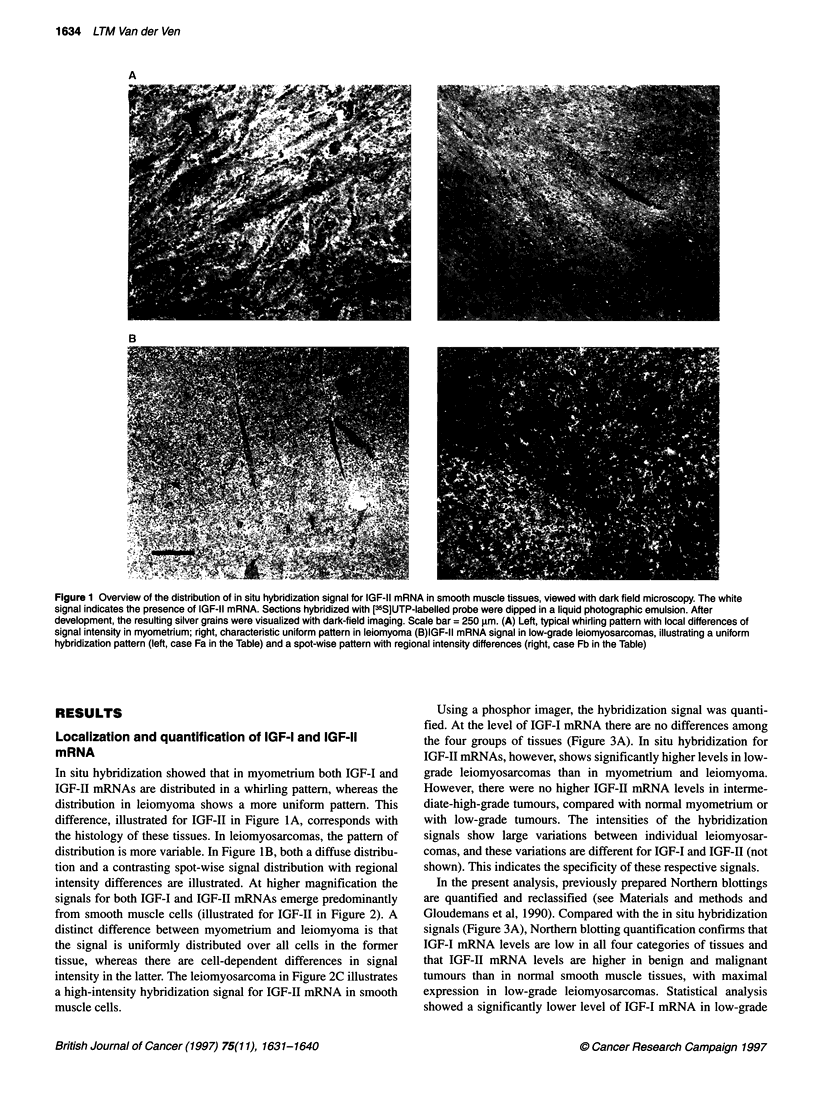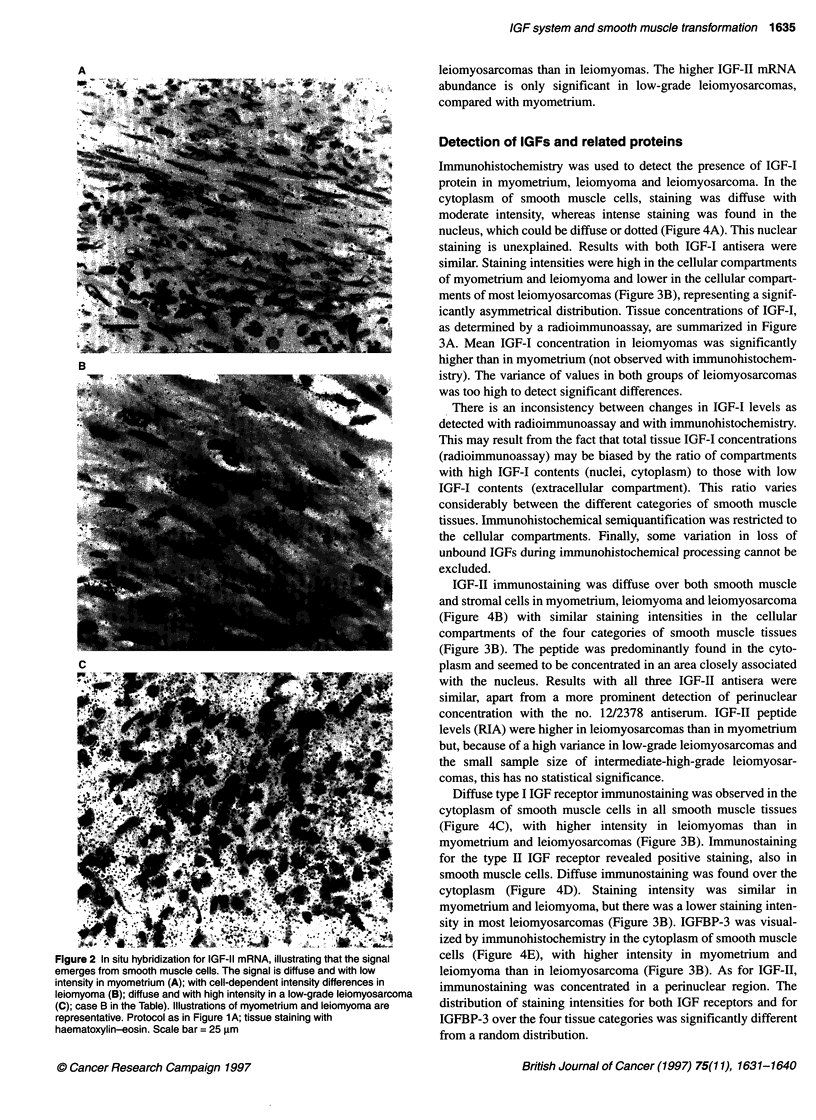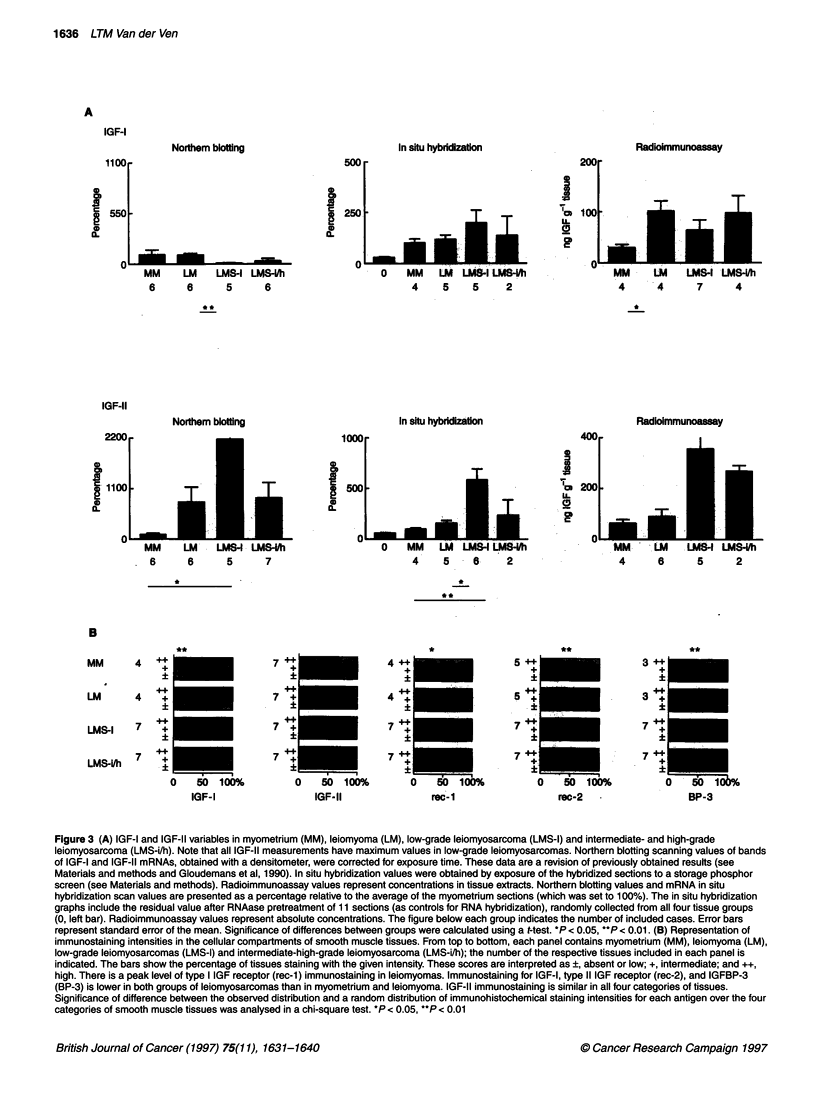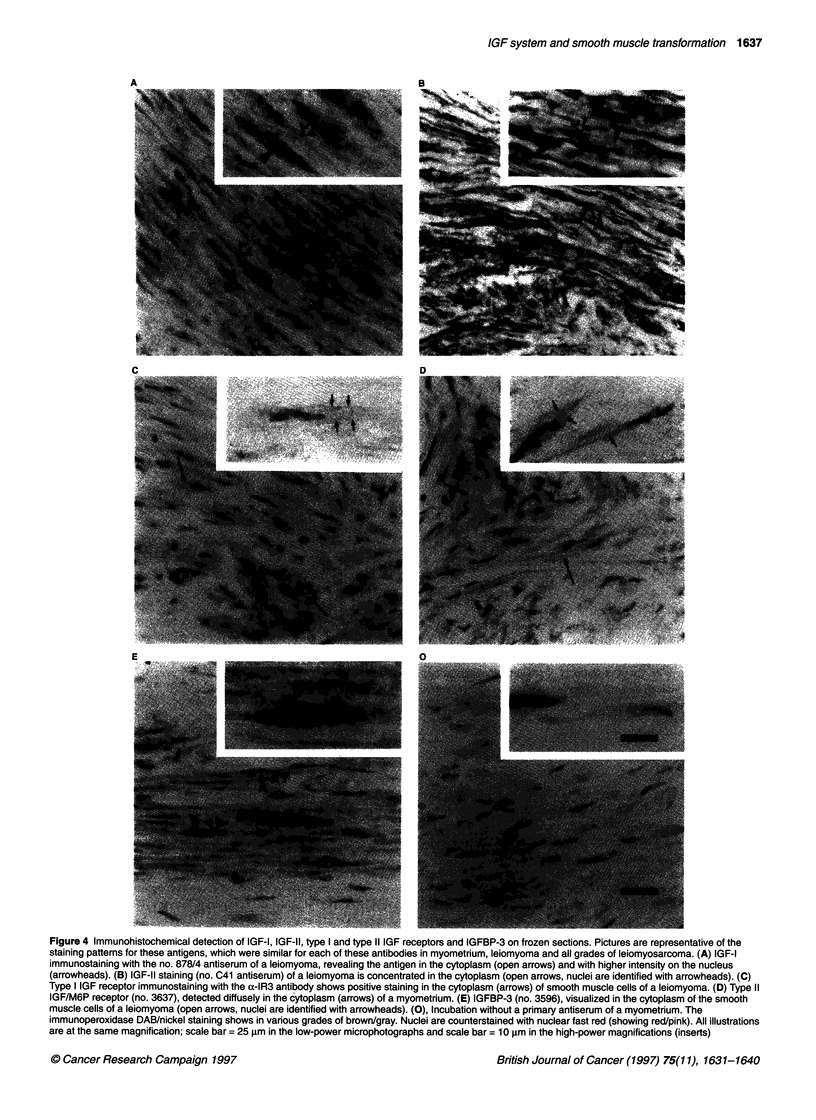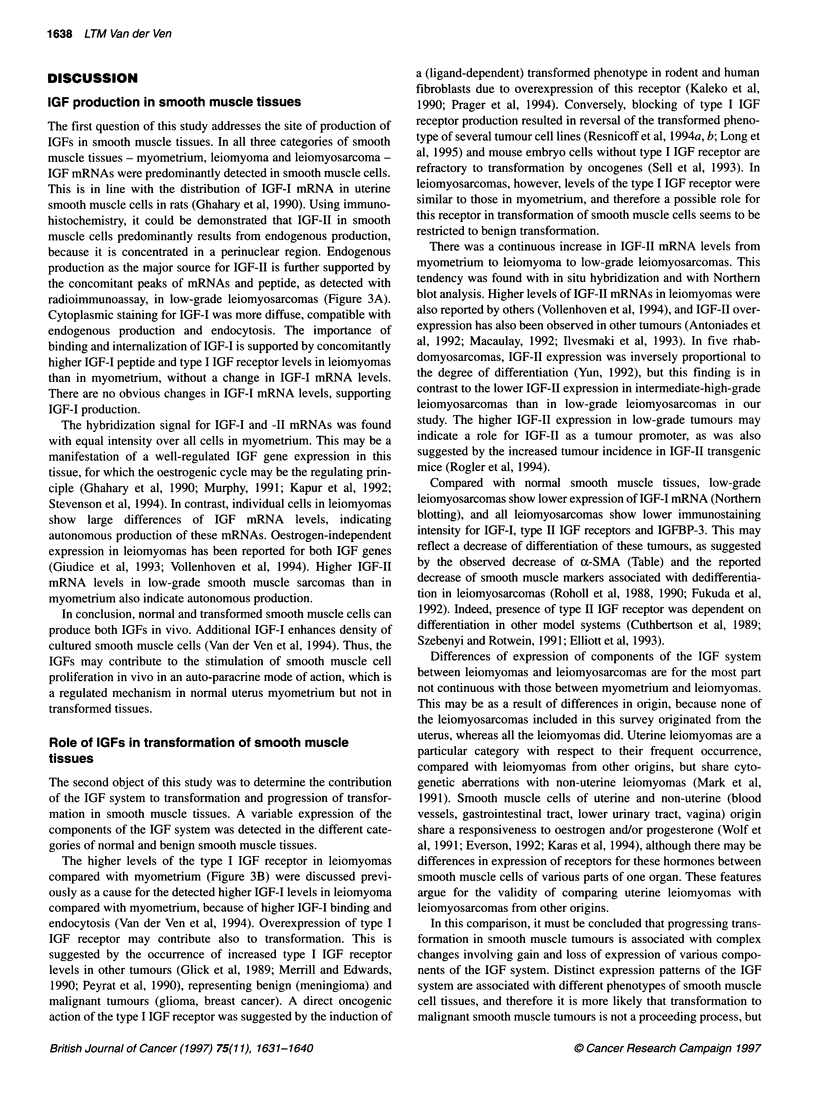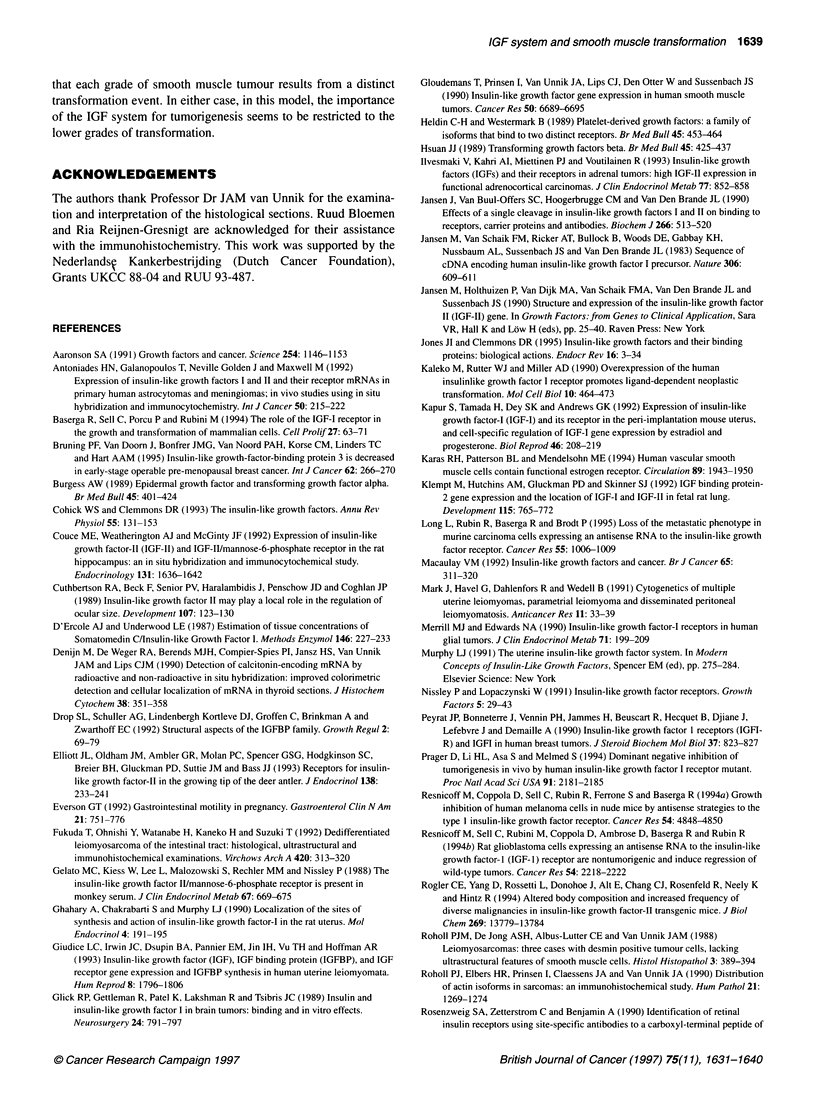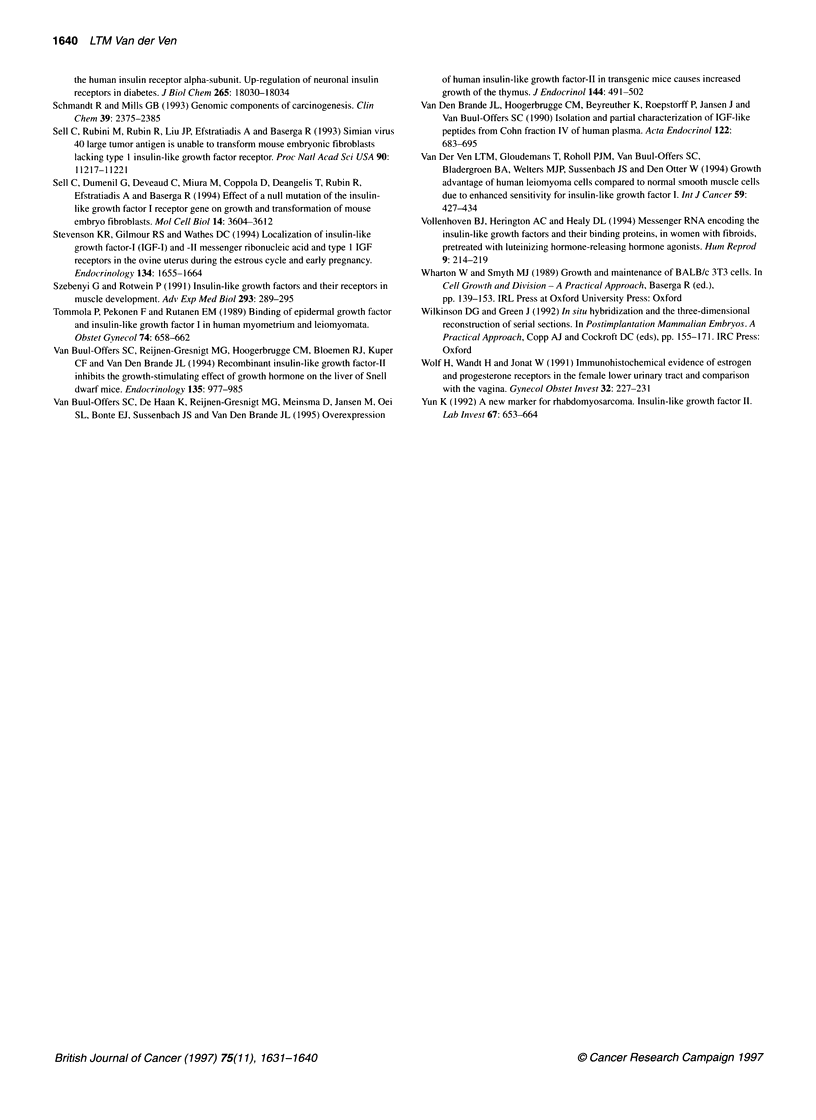# Expression of insulin-like growth factors (IGFs), their receptors and IGF binding protein-3 in normal, benign and malignant smooth muscle tissues.

**DOI:** 10.1038/bjc.1997.278

**Published:** 1997

**Authors:** L. T. Van der Ven, P. J. Roholl, T. Gloudemans, S. C. Van Buul-Offers, M. J. Welters, B. A. Bladergroen, J. A. Faber, J. S. Sussenbach, W. Den Otter

**Affiliations:** Department of Functional Morphology, Veterinary Faculty Utrecht University, The Netherlands.

## Abstract

**Images:**


British Journal of Cancer (1997) 75(11), 1631–1640
© 1997 Cancer Research Campaign

### Expression of insulin-like growth factors (IGFs), their receptors and IGF binding protein-3 in normal, benign and malignant smooth muscle tissues

**LTM Van der Ven[1], PJM Roholl[2], T Gloudemans[3], SC Van Buul-Offers[4], MJP Welters[1], BA Bladergroen[1], JAJ Faber[5], JS Sussenbach[3] and W Den Otter[1]**

[1]Department of Functional Morphology, Veterinary Faculty Utrecht University, PO Box 80.157, NL-3508 TD Utrecht, The Netherlands; [2]Laboratory for Pathology, National Institute for Public Health and Environmental Protection (RIVM), PO Box 1, NL-3720 BA Bilthoven, The Netherlands; [3]Laboratory for Physiological Chemistry, Utrecht University, PO Box 80030, NL-3508 TA Utrecht, The Netherlands; [4]Department of Endocrinology, Wilhelmina Children's Hospital, University of Utrecht, PO Box 18009, NL-3501 CA Utrecht, The Netherlands; [5]Biostatistical Centre, Utrecht University, Padualaan 14, NL-3584 CH, The Netherlands

**Summary** To assess the role of insulin-like growth factors (IGFs) in growth and transformation of normal (myometrium) and tumorous smooth muscle cell (SMC) tissues, in situ hybridization (ISH) analysis for insulin-like growth factor I and II (IGF-I and IGF-II) mRNAs was combined with detection of IGF peptides, their receptors and IGF binding protein-3 (IGFBP-3). mRNAs for both IGFs were detected in smooth muscle cells in normal, benign and malignant SMC tissues, together with the IGF peptides, both IGF receptors and IGFBP-3. This suggests an autocrine role for both IGFs. Leiomyomas had higher IGF-I peptide levels and higher levels of type I IGF receptors than myometrium, supporting the idea that IGFs play a role in the growth and transformation of these tumours. Low-grade leiomyosarcomas contained more IGF-II mRNAs than myometrium and leiomyoma, fewer type II IGF/mannose 6-phosphate receptors and less IGFBP-3 than myometrium and, in addition, fewer IGF-I mRNAs and type I IGF receptors than leiomyoma. Intermediate- and high-grade leiomyosarcomas had intermediate levels of IGF-II mRNAs and peptide, ranging between those in myometrium and low-grade leiomyosarcomas. Thus, growth and transformation of leiomyosarcomas may be regulated by IGF-II, although more markedly in low-grade than in high-grade leiomyosarcomas. In conclusion, the various categories of SMC tissues are associated with a distinct expression pattern of the IGF system. This suggests that each category of SMC tumours arises as a distinct entity and that there is no progression of transformation in these tissues.

**Keywords:** insulin-like growth factors; carcinogenesis; binding proteins; receptors; smooth muscle cells; transformation

Transformation is a stepwise process in which cells proceed to a different phenotype because of genomic alterations. At the functional level, the transformed phenotype is characterized by one or more of the following features: immortalization, change in cell morphology, in vitro focus formation due to decreased contact inhibition, reduced in vitro requirement of serum growth factors, anchorage-independent growth (growth in soft agar) and in vivo tumorigenicity (Wharton and Smyth, 1989). Transformation requires the activation of specific transformation signalling pathways. This is illustrated by the observation that type I insulin-like growth factor receptor-deficient fibroblasts, which have impaired growth capacity, acquire a transformed phenotype after transfection with *ras*, without reversal of the growth deficiencies (Baserga et al, 1994; Sell et al, 1994). Thus, transformation is independent of increased cell proliferation, although unrestricted proliferation may be favoured with progressing transformation. Similarly, apoptosis and transformation are independent functions, although the apoptosis-inducing pathway may be blocked by the accumulation of genomic alterations, e.g. by the activation of the apoptosis blocking oncogene *bcl2* (Aaronson, 1991; Schmandt and Mills, 1993).

*Received 23 May 1996*
*Revised 29 October 1996*
*Accepted 14 November 1996*

*Correspondence to:* LTM Van der Ven

Initiation of transformation may result from deregulated activity of any factor in transformation signalling pathways and thus may involve overstimulation with growth factors, enhanced receptor signalling and increased activity of second messengers and of nuclear binding proteins. Transforming growth factor $\alpha$ and $\beta$ and platelet-derived growth factor BB are typical inducers of transformation in selected systems (Burgess, 1989; Heldin and Westermark, 1989; Hsuan, 1989). In addition, transformation may result from decreased activity of transformation-inhibiting factors, known as products of anti-oncogenes or tumour-suppressor genes (Schmandt and Mills, 1993).

The insulin-like growth factors (IGF-I and IGF-II) are polypeptide growth factors, which play an important role in cellular and somatic growth (Cohick and Clemmons, 1993). Both IGFs can bind to the type I IGF receptor with similar affinity, whereas the type II IGF/mannose 6-phosphate receptor preferentially binds IGF-II. The type I IGF receptor mediates the mitogenic action of both IGFs (Nissley and Lopaczynski, 1991), and the type II IGF receptor is mainly associated with differentiation and histomorphogenesis (Nissley and Lopaczynski, 1991). IGF functions are modulated by IGF-binding proteins (IGFBPs), of which six distinct variants have been characterized (Drop et al, 1992). At the cellular level, these IGFBPs may facilitate the binding of IGFs to their receptors or they may inhibit this binding (Jones and Clemmons, 1995). Although IGFs are not designated as (proto)oncogene products, there are many indications that they have transformation-inducing capacity. For instance, overexpression of the IGFs or of the type I IGF receptor is observed in many human tumours (Glick et al, 1989; Tommola et al, 1989; Merrill and Edwards, 1990; Macaulay, 1992).

We have previously shown that higher IGF-I concentrations in leiomyoma than in myometrium may be as a result of the higher levels of type I IGF receptors in leiomyoma (Van der Ven et al, 1994). The increased responsiveness to IGF-I of cultured leiomyoma smooth muscle cells compared with their normal counterparts is in line with this observation. Furthermore, malignant transformation of smooth muscle tissues is associated with increased expression of IGF-II (Gloudemans et al, 1990). In the present study, employing in situ hybridization for IGF-I mRNA, we analyse whether the increased levels of IGF-I in benign compared with normal smooth muscle cells could be of autocrine source. This series is completed with an analysis of malignant smooth muscle tissues. In these tissues, we also compare IGF-II mRNA hybridization signal, IGF-I and -II peptide concentrations, immunohistochemical localizations and staining intensities, and immunohistochemistry of both IGF receptors and of IGFBP-3 to find indications for a role of the IGF system during oncogenic transformation in these tissues.

### MATERIALS AND METHODS

### Tissue collection

Normal and benign smooth muscle tissues were obtained from the uteri of patients that had undergone hysterectomy to treat leiomyoma. Informed consent was obtained according to the guidelines of the Dutch Cancer Foundation and the Ethical Council of the Utrecht University Hospital. Leiomyosarcomas were resected from a variety of organs. Specimens were collected from surgery as soon as possible. Part of the tissue was snap-frozen in liquid nitrogen and stored at −80°C for further use in mRNA or peptide extraction procedures and for immunohistochemistry. Another part was routinely fixed in 4% phosphate-buffered formaldehyde (Klinipath, Duiven, The Netherlands), dehydrated through a graded ethanol/xylene series and embedded in paraffin. Data of the tissues that were included in this analysis are outlined in the Table. Most of the included tissues show positive staining for α-smooth muscle actin or desmin, confirming the smooth muscle phenotype. Loss of expression of smooth muscle markers in leiomyosarcomas is a common event in these tumours (Roholl et al, 1990). The shown percentage of necrosis was estimated on histological sections. A high necrotic index would bias the results from radioimmunoassays. Therefore, tumours with a high percentage of necrosis (> 50%) were excluded from assays in which vitality of the tissue could not be checked. The protocol for use of the tissues in this study was approved by the Ethical Council of the Utrecht University Hospital.

### Northern blotting analysis

The presented Northern blotting data are a revision of previous results (Gloudemans et al, 1990). The original films were scanned in a densitometer, and scanning values of 7.6-kb IGF-I mRNA and of 4.8- and 6.0-kb IGF-II mRNA bands were corrected for exposure time. The Northern blotting analysis comprises the same tissue cohort, although more samples were processed for Northern blotting than for in situ hybridization. In the present analysis, the quantified Northern blotting results are rearranged according to the classification shown in the Table.

### In situ hybridization

From the paraffinized tissues, 4-μm sections were prepared on aminopropyltriethoxysilane (Sigma Chemicals, St Louis, MO, USA) coated slides. Specific mRNAs were detected by subjecting the sections to an in situ hybridization procedure (Denijn et al,

**Table** Clinical data and characteristics of tissues

| Code | Sex, age (years) | Site of resection | Tissue | Smooth muscle markers[a] | | Necrosis (%) |
|---|---|---|---|---|---|---|
| | | | | α-SMA | Desmin | |
| *Normal myometrium (MM) and leiomyoma (LM)* | | | | | | |
| A–G | F, 38–50 | Uterus | Normal MM, LM | All ++ | | 0 |
| *Leiomyosarcoma* | | | *Grading*[b] | | | |
| A | M, 27 | Oesophagus | Low | ++ | | 0 |
| B | M, 69 | Stomach | Low | – | + | 0 |
| C | M, 78 | Stomach | Low | + | – | < 10 |
| D | F, 73 | Stomach | Low | ++ | | < 10 |
| E | M, 32 | Retroperitoneal | Low | ++ | | < 5 |
| Fa[c] | F, 34 | Mesenteric lymph node | Low | – | ± | 0 |
| Fb | F, 38 | Abdominal wall metastasis | Low | + | + | < 10 |
| G | M, 41 | Mesenterium | Intermediate | + | + | < 10 |
| H | M, 82 | Subcutis (left arm) | Intermediate | ++ | + | 10–50 |
| I | M, 55 | Retroperitoneal | Intermediate | + | | > 50 |
| J | M, 69 | Right axilla | Intermediate | + | + | > 50 |
| K | F, 70 | M. quadriceps femoris | Intermediate | – | | < 10 |
| La[d] | F, 53 | Retroperitoneum (recurrence) | High | ++ | | 10–50 |
| Lb | F, 53 | Retroperitoneum (recurrence) | High | – | | > 50 |

[a]Additional phenotyping was done with immunohistochemical staining for the smooth muscle markers α-SMA and desmin, which are scored in a range from –, no staining to ++, high-intensity staining. Blanks in the 'desmin' column are not tested. [b]Leiomyosarcomas were graded according to the number of mitoses per mm$^2$ (< 3, low grade; 4–25, intermediate grade; and > 25, high grade) and are arranged with increasing mitotic index. [c]Tumours Fa and Fb originated from the same patient with an interval of 3.5 years. [d]Tumours La and Lb originated from the same patient with an interval of 8.5 months.

 © Cancer Research Campaign 1997

1990; Wilkinson and Green, 1992) with antisense RNA probes for IGF-I and IGF-II. These were prepared from cDNA of IGF-I (pIGF-I, exons 1, 3 and 4; 777 base pairs; Jansen et al, 1983) and cDNA of IGF-II (pIGF-IIvar, exons 3, 7, 8 and 9; 713 base pairs; Jansen M et al, 1990), cloned in the vector pBluescript-KS (Stratagene, La Jolla, CA, USA) and reverse transcribed with T3 RNA polymerase (Boehringer, Mannheim, Germany) in the presence of [$^{35}$S]UTP (Amersham, Amersham, UK). Specificity of these probes was tested in tissue sections from mice transgenic for human IGF-II (Van Buul-Offers et al, 1995) and, furthermore, IGF-I and IGF-II hybridization signals were compared with the signal of a thrombin receptor probe on smooth muscle tissue sections. Briefly, the in situ hybridization procedure includes dewaxing, rehydration, permeabilization in Triton X-100 (Boehringer), treatment with 50 µg ml$^{-1}$ proteinase K (Boehringer), acetylation and dehydration of the sections. Control sections were subjected to a RNAase treatment (100 µg ml$^{-1}$ RNAase A and 1 µg ml$^{-1}$ RNAase T1; both from Boehringer) to degrade the hybridization target. Labelled probe in a concentration of 200 000 c.p.m. per 30 µl of hybridization buffer was incubated with the sections overnight at 55°C and, after several washing steps, sections were dehydrated in a series of ethanol containing 0.3 M ammonium acetate, air-dried and exposed to a storage phosphor screen (Molecular Dynamics) for 8–18 h, depending on the signal intensity. This screen was scanned with a Phosphor Imager (Molecular Dynamics), and the signal of a representative, standardized surface of approximately 0.4 cm$^2$ was quantified. Data from individual sections were transformed to a percentage relative to the mean of the four scanned myometrium sections.

### Immunohistochemistry

Tissue cryosections of 6 µm were prepared with a microtome-cryostat (Damon IEC, Needham, MA, USA) on gelatin-coated slides and air-dried. These sections were fixed with paraformaldehyde (Merck, Darmstadt, Germany) and subjected to an immunocytochemical procedure with specific polyclonal antisera for human IGF-I (batches no. 874 diluted 1:150 and no. 878/4 diluted 1:250, which were kind gifts from Dr BH Breier, Auckland, New Zealand); for human IGF-II (batches no. C41 and no. C65, diluted 1:200, also obtained from Drs BH Breier and PD Gluckman; and batch no. 12/2378, diluted 1:100, a kind gift of Dr J Zapf, Zürich, Switzerland); for the human type I IGF receptor, diluted 1:100 (a kind gift of Dr SA Rosenzweig, New Haven, CO, USA); for the rat type II IGF/M6P receptor, diluted 1:2000 (batch no. 3637, a kind gift of Dr SP Nissley, Bethesda, MD, USA); for human IGFBP-3, batch no. 3596, diluted 1:1000 (a generous gift of Dr J Van Doorn, Utrecht, The Netherlands); and monoclonal antibodies for the human type I IGF receptor, dilution 1:25 (α-IR3; Oncogene Science, Manhassat, NY, USA); for α-smooth muscle actin, dilution 1:400 (α-SMA, Sigma); and a polyclonal antiserum for desmin (Eurodiagnostics, Apeldoorn, The Netherlands). The last two antibodies were included as independent phenotypic markers. Antisera no. 878/4 and no. C65 were characterized for use in immunohistochemistry by Klempt et al (1992); specificity for immunostaining of the polyclonal antisera for the type I and type II IGF receptors was demonstrated by Rosenzweig et al (1990) and by Couce et al (1992) respectively. The no. 3637 antiserum cross-reacts with the human type II IGF receptor (Gelato et al, 1988). The IGFBP-3 antiserum recognizes the IGFBP-3 38.5–41.5-kDa bands and IGFBP-3 degradation products in an immunoblotting of human serum, but not IGFBP-2 and -4. In a radioimmunoassay, there is no cross-reactivity of the IGFBP-3 antiserum to IGFBP-1. Binding of IGFs to IGFBP-3 does not interfere with binding of the antiserum to IGFBP-3 (Bruning et al, 1995). Preincubation of the IGFBP-3 antiserum with its antigen induced a reduction of immunostaining intensity of 50–90% in various normal and transformed smooth muscle tissues. Background staining was checked by omitting the primary antibody. The immunohistochemical procedure was described elsewhere (Van der Ven et al, 1994) and includes preincubation with a normal serum, analogous to the species origin of the second antibody, incubation with the primary antibody and an appropriate biotinylated second antibody (together with the normal sera obtained from Vector, Burlingame, CA, USA). These steps were alternated with washes with phosphate-buffered saline (PBS)/Tween. The bound immune complex was visualized with horseradish peroxidase–avidin–biotin complex (Vector). As a chromogenic substrate for the horseradish peroxidase, we used nickel-enhanced 3,3′diaminobenzidine (DAB, Merck). Nuclei were counterstained with nuclear fast red. Immunostained cells were dehydrated in ethanol/xylene and embedded in DePeX. Immunostained sections were examined by two independent observers for staining pattern (appearance and localization) and for staining intensity. Each independent antibody was scored subjectively in a range including absent or low (±), intermediate (+) and high (++) staining intensity. For each variable, results from duplicate observations with either the same or different antisera were pooled.

### Radioimmunoassay (RIA)

For determination of IGF-I and IGF-II concentrations in the smooth muscle tissues, tissue extracts were prepared as described previously (D'Ercole and Underwood, 1987; Van der Ven et al, 1994). IGF-binding proteins were eliminated from these extracts under acid conditions using $C_{18}$ Sep-Pak cartridges (Sep-Pak, Waters, Milford, MA, USA; Van der Ven et al, 1994), and IGFs were measured in a radioimmunoassay as described previously (Jansen J et al, 1990; Van Buul-Offers et al, 1994; Van der Ven et al, 1994). The results were expressed in ng ml$^{-1}$, using recombinant IGF-I (International Reference Reagent 87/518 from the National Institute for Biological Standards and Control, Potters Bar, Herts, UK) and native human IGF-II extracted in our own laboratory (Van den Brande et al, 1990) as the reference peptides respectively. For the IGF-I RIA, the intra- and interassay coefficients of variation were 5.9% and 7.9%, and for the IGF-II RIA these values were 7.0% and 7.7% (Bruning et al, 1995). The lower detection limit was 0.005 ng per tube for IGF-I and 0.014 ng per tube for IGF-II.

### Statistical analysis

Differences of levels of mRNAs and peptides of IGF-I and IGF-II among the four categories of tissues (normal, benign, low-grade malignant and intermediate-high-grade malignant) were tested for significance using a Student's *t*-test or in a Welch *t*-test when variances between groups departed significantly from homogeneity. Significance of difference of the observed distribution from a random distribution of immunohistochemical staining intensities for IGF-I, IGF-II, both IGF receptors or IGFBP-3 over the four categories of smooth muscle tissues was analysed in a chi-square test.

© Cancer Research Campaign 1997

A

B

**Figure 1** Overview of the distribution of in situ hybridization signal for IGF-II mRNA in smooth muscle tissues, viewed with dark field microscopy. The white signal indicates the presence of IGF-II mRNA. Sections hybridized with [$^{35}$S]UTP-labelled probe were dipped in a liquid photographic emulsion. After development, the resulting silver grains were visualized with dark-field imaging. Scale bar = 250 μm. (**A**) Left, typical whirling pattern with local differences of signal intensity in myometrium; right, characteristic uniform pattern in leiomyoma (B)IGF-II mRNA signal in low-grade leiomyosarcomas, illustrating a uniform hybridization pattern (left, case Fa in the Table) and a spot-wise pattern with regional intensity differences (right, case Fb in the Table)

### RESULTS

### Localization and quantification of IGF-I and IGF-II mRNA

In situ hybridization showed that in myometrium both IGF-I and IGF-II mRNAs are distributed in a whirling pattern, whereas the distribution in leiomyoma shows a more uniform pattern. This difference, illustrated for IGF-II in Figure 1A, corresponds with the histology of these tissues. In leiomyosarcomas, the pattern of distribution is more variable. In Figure 1B, both a diffuse distribution and a contrasting spot-wise signal distribution with regional intensity differences are illustrated. At higher magnification the signals for both IGF-I and IGF-II mRNAs emerge predominantly from smooth muscle cells (illustrated for IGF-II in Figure 2). A distinct difference between myometrium and leiomyoma is that the signal is uniformly distributed over all cells in the former tissue, whereas there are cell-dependent differences in signal intensity in the latter. The leiomyosarcoma in Figure 2C illustrates a high-intensity hybridization signal for IGF-II mRNA in smooth muscle cells.

Using a phosphor imager, the hybridization signal was quantified. At the level of IGF-I mRNA there are no differences among the four groups of tissues (Figure 3A). In situ hybridization for IGF-II mRNAs, however, shows significantly higher levels in low-grade leiomyosarcomas than in myometrium and leiomyoma. However, there were no higher IGF-II mRNA levels in intermediate-high-grade tumours, compared with normal myometrium or with low-grade tumours. The intensities of the hybridization signals show large variations between individual leiomyosarcomas, and these variations are different for IGF-I and IGF-II (not shown). This indicates the specificity of these respective signals.

In the present analysis, previously prepared Northern blottings are quantified and reclassified (see Materials and methods and Gloudemans et al, 1990). Compared with the in situ hybridization signals (Figure 3A), Northern blotting quantification confirms that IGF-I mRNA levels are low in all four categories of tissues and that IGF-II mRNA levels are higher in benign and malignant tumours than in normal smooth muscle tissues, with maximal expression in low-grade leiomyosarcomas. Statistical analysis showed a significantly lower level of IGF-I mRNA in low-grade

© Cancer Research Campaign 1997

**Figure 2** In situ hybridization for IGF-II mRNA, illustrating that the signal emerges from smooth muscle cells. The signal is diffuse and with low intensity in myometrium (**A**); with cell-dependent intensity differences in leiomyoma (**B**); diffuse and with high intensity in a low-grade leiomyosarcoma (**C**); case B in the Table). Illustrations of myometrium and leiomyoma are representative. Protocol as in Figure 1A; tissue staining with haematoxylin–eosin. Scale bar = 25 μm leiomyosarcomas than in leiomyomas. The higher IGF-II mRNA abundance is only significant in low-grade leiomyosarcomas, compared with myometrium.

### Detection of IGFs and related proteins

Immunohistochemistry was used to detect the presence of IGF-I protein in myometrium, leiomyoma and leiomyosarcoma. In the cytoplasm of smooth muscle cells, staining was diffuse with moderate intensity, whereas intense staining was found in the nucleus, which could be diffuse or dotted (Figure 4A). This nuclear staining is unexplained. Results with both IGF-I antisera were similar. Staining intensities were high in the cellular compartments of myometrium and leiomyoma and lower in the cellular compartments of most leiomyosarcomas (Figure 3B), representing a significantly asymmetrical distribution. Tissue concentrations of IGF-I, as determined by a radioimmunoassay, are summarized in Figure 3A. Mean IGF-I concentration in leiomyomas was significantly higher than in myometrium (not observed with immunohistochemistry). The variance of values in both groups of leiomyosarcomas was too high to detect significant differences.

There is an inconsistency between changes in IGF-I levels as detected with radioimmunoassay and with immunohistochemistry. This may result from the fact that total tissue IGF-I concentrations (radioimmunoassay) may be biased by the ratio of compartments with high IGF-I contents (nuclei, cytoplasm) to those with low IGF-I contents (extracellular compartment). This ratio varies considerably between the different categories of smooth muscle tissues. Immunohistochemical semiquantification was restricted to the cellular compartments. Finally, some variation in loss of unbound IGFs during immunohistochemical processing cannot be excluded.

IGF-II immunostaining was diffuse over both smooth muscle and stromal cells in myometrium, leiomyoma and leiomyosarcoma (Figure 4B) with similar staining intensities in the cellular compartments of the four categories of smooth muscle tissues (Figure 3B). The peptide was predominantly found in the cytoplasm and seemed to be concentrated in an area closely associated with the nucleus. Results with all three IGF-II antisera were similar, apart from a more prominent detection of perinuclear concentration with the no. 12/2378 antiserum. IGF-II peptide levels (RIA) were higher in leiomyosarcomas than in myometrium but, because of a high variance in low-grade leiomyosarcomas and the small sample size of intermediate-high-grade leiomyosarcomas, this has no statistical significance.

Diffuse type I IGF receptor immunostaining was observed in the cytoplasm of smooth muscle cells in all smooth muscle tissues (Figure 4C), with higher intensity in leiomyomas than in myometrium and leiomyosarcomas (Figure 3B). Immunostaining for the type II IGF receptor revealed positive staining, also in smooth muscle cells. Diffuse immunostaining was found over the cytoplasm (Figure 4D). Staining intensity was similar in myometrium and leiomyoma, but there was a lower staining intensity in most leiomyosarcomas (Figure 3B). IGFBP-3 was visualized by immunohistochemistry in the cytoplasm of smooth muscle cells (Figure 4E), with higher intensity in myometrium and leiomyoma than in leiomyosarcoma (Figure 3B). As for IGF-II, immunostaining was concentrated in a perinuclear region. The distribution of staining intensities for both IGF receptors and for IGFBP-3 over the four tissue categories was significantly different from a random distribution.

*© Cancer Research Campaign 1997*

**Figure 3** (**A**) IGF-I and IGF-II variables in myometrium (MM), leiomyoma (LM), low-grade leiomyosarcoma (LMS-I) and intermediate- and high-grade leiomyosarcoma (LMS-i/h). Note that all IGF-II measurements have maximum values in low-grade leiomyosarcomas. Northern blotting scanning values of bands of IGF-I and IGF-II mRNAs, obtained with a densitometer, were corrected for exposure time. These data are a revision of previously obtained results (see Materials and methods and Gloudemans et al, 1990). In situ hybridization values were obtained by exposure of the hybridized sections to a storage phosphor screen (see Materials and methods). Radioimmunoassay values represent concentrations in tissue extracts. Northern blotting values and mRNA in situ hybridization scan values are presented as a percentage relative to the average of the myometrium sections (which was set to 100%). The in situ hybridization graphs include the residual value after RNAase pretreatment of 11 sections (as controls for RNA hybridization), randomly collected from all four tissue groups (0, left bar). Radioimmunoassay values represent absolute concentrations. The figure below each group indicates the number of included cases. Error bars represent standard error of the mean. Significance of differences between groups were calculated using a *t*-test. $^{*}P < 0.05$, $^{**}P < 0.01$. (**B**) Representation of immunostaining intensities in the cellular compartments of smooth muscle tissues. From top to bottom, each panel contains myometrium (MM), leiomyoma (LM), low-grade leiomyosarcomas (LMS-I) and intermediate-high-grade leiomyosarcoma (LMS-i/h); the number of the respective tissues included in each panel is indicated. The bars show the percentage of tissues staining with the given intensity. These scores are interpreted as ±, absent or low; +, intermediate; and ++, high. There is a peak level of type I IGF receptor (rec-1) immunostaining in leiomyomas. Immunostaining for IGF-I, type II IGF receptor (rec-2), and IGFBP-3 (BP-3) is lower in both groups of leiomyosarcomas than in myometrium and leiomyoma. IGF-II immunostaining is similar in all four categories of tissues. Significance of difference between the observed distribution and a random distribution of immunohistochemical staining intensities for each antigen over the four categories of smooth muscle tissues was analysed in a chi-square test. $^{*}P < 0.05$, $^{**}P < 0.01$

 © Cancer Research Campaign 1997

**Figure 4** Immunohistochemical detection of IGF-I, IGF-II, type I and type II IGF receptors and IGFBP-3 on frozen sections. Pictures are representative of the staining patterns for these antigens, which were similar for each of these antibodies in myometrium, leiomyoma and all grades of leiomyosarcoma. (**A**) IGF-I immunostaining with the no. 878/4 antiserum of a leiomyoma, revealing the antigen in the cytoplasm (open arrows) and with higher intensity on the nucleus (arrowheads). (**B**) IGF-II staining (no. C41 antiserum) of a leiomyoma is concentrated in the cytoplasm (open arrows, nuclei are identified with arrowheads). (**C**) Type I IGF receptor immunostaining with the α-IR3 antibody shows positive staining in the cytoplasm (arrows) of smooth muscle cells of a leiomyoma. (**D**) Type II IGF/M6P receptor (no. 3637), detected diffusely in the cytoplasm (arrows) of a myometrium. (**E**) IGFBP-3 (no. 3596), visualized in the cytoplasm of the smooth muscle cells of a leiomyoma (open arrows, nuclei are identified with arrowheads). (**O**), Incubation without a primary antiserum of a myometrium. The immunoperoxidase DAB/nickel staining shows in various grades of brown/gray. Nuclei are counterstained with nuclear fast red (showing red/pink). All illustrations are at the same magnification; scale bar = 25 μm in the low-power microphotographs and scale bar = 10 μm in the high-power magnifications (inserts)

© Cancer Research Campaign 1997

### DISCUSSION

### IGF production in smooth muscle tissues

The first question of this study addresses the site of production of IGFs in smooth muscle tissues. In all three categories of smooth muscle tissues – myometrium, leiomyoma and leiomyosarcoma – IGF mRNAs were predominantly detected in smooth muscle cells. This is in line with the distribution of IGF-I mRNA in uterine smooth muscle cells in rats (Ghahary et al, 1990). Using immunohistochemistry, it could be demonstrated that IGF-II in smooth muscle cells predominantly results from endogenous production, because it is concentrated in a perinuclear region. Endogenous production as the major source for IGF-II is further supported by the concomitant peaks of mRNAs and peptide, as detected with radioimmunoassay, in low-grade leiomyosarcomas (Figure 3A). Cytoplasmic staining for IGF-I was more diffuse, compatible with endogenous production and endocytosis. The importance of binding and internalization of IGF-I is supported by concomitantly higher IGF-I peptide and type I IGF receptor levels in leiomyomas than in myometrium, without a change in IGF-I mRNA levels. There are no obvious changes in IGF-I mRNA levels, supporting IGF-I production.

The hybridization signal for IGF-I and -II mRNAs was found with equal intensity over all cells in myometrium. This may be a manifestation of a well-regulated IGF gene expression in this tissue, for which the oestrogenic cycle may be the regulating principle (Ghahary et al, 1990; Murphy, 1991; Kapur et al, 1992; Stevenson et al, 1994). In contrast, individual cells in leiomyomas show large differences of IGF mRNA levels, indicating autonomous production of these mRNAs. Oestrogen-independent expression in leiomyomas has been reported for both IGF genes (Giudice et al, 1993; Vollenhoven et al, 1994). Higher IGF-II mRNA levels in low-grade smooth muscle sarcomas than in myometrium also indicate autonomous production.

In conclusion, normal and transformed smooth muscle cells can produce both IGFs in vivo. Additional IGF-I enhances density of cultured smooth muscle cells (Van der Ven et al, 1994). Thus, the IGFs may contribute to the stimulation of smooth muscle cell proliferation in vivo in an auto-paracrine mode of action, which is a regulated mechanism in normal uterus myometrium but not in transformed tissues.

### Role of IGFs in transformation of smooth muscle tissues

The second object of this study was to determine the contribution of the IGF system to transformation and progression of transformation in smooth muscle tissues. A variable expression of the components of the IGF system was detected in the different categories of normal and benign smooth muscle tissues.

The higher levels of the type I IGF receptor in leiomyomas compared with myometrium (Figure 3B) were discussed previously as a cause for the detected higher IGF-I levels in leiomyoma compared with myometrium, because of higher IGF-I binding and endocytosis (Van der Ven et al, 1994). Overexpression of type I IGF receptor may contribute also to transformation. This is suggested by the occurrence of increased type I IGF receptor levels in other tumours (Glick et al, 1989; Merrill and Edwards, 1990; Peyrat et al, 1990), representing benign (meningioma) and malignant tumours (glioma, breast cancer). A direct oncogenic action of the type I IGF receptor was suggested by the induction of a (ligand-dependent) transformed phenotype in rodent and human fibroblasts due to overexpression of this receptor (Kaleko et al, 1990; Prager et al, 1994). Conversely, blocking of type I IGF receptor production resulted in reversal of the transformed phenotype of several tumour cell lines (Resnicoff et al, 1994*a*, *b*; Long et al, 1995) and mouse embryo cells without type I IGF receptor are refractory to transformation by oncogenes (Sell et al, 1993). In leiomyosarcomas, however, levels of the type I IGF receptor were similar to those in myometrium, and therefore a possible role for this receptor in transformation of smooth muscle cells seems to be restricted to benign transformation.

There was a continuous increase in IGF-II mRNA levels from myometrium to leiomyoma to low-grade leiomyosarcomas. This tendency was found with in situ hybridization and with Northern blot analysis. Higher levels of IGF-II mRNAs in leiomyomas were also reported by others (Vollenhoven et al, 1994), and IGF-II overexpression has also been observed in other tumours (Antoniades et al, 1992; Macaulay, 1992; Ilvesmaki et al, 1993). In five rhabdomyosarcomas, IGF-II expression was inversely proportional to the degree of differentiation (Yun, 1992), but this finding is in contrast to the lower IGF-II expression in intermediate-high-grade leiomyosarcomas than in low-grade leiomyosarcomas in our study. The higher IGF-II expression in low-grade tumours may indicate a role for IGF-II as a tumour promoter, as was also suggested by the increased tumour incidence in IGF-II transgenic mice (Rogler et al, 1994).

Compared with normal smooth muscle tissues, low-grade leiomyosarcomas show lower expression of IGF-I mRNA (Northern blotting), and all leiomyosarcomas show lower immunostaining intensity for IGF-I, type II IGF receptors and IGFBP-3. This may reflect a decrease of differentiation of these tumours, as suggested by the observed decrease of α-SMA (Table) and the reported decrease of smooth muscle markers associated with dedifferentiation in leiomyosarcomas (Roholl et al, 1988, 1990; Fukuda et al, 1992). Indeed, presence of type II IGF receptor was dependent on differentiation in other model systems (Cuthbertson et al, 1989; Szebenyi and Rotwein, 1991; Elliott et al, 1993).

Differences of expression of components of the IGF system between leiomyomas and leiomyosarcomas are for the most part not continuous with those between myometrium and leiomyomas. This may be as a result of differences in origin, because none of the leiomyosarcomas included in this survey originated from the uterus, whereas all the leiomyomas did. Uterine leiomyomas are a particular category with respect to their frequent occurrence, compared with leiomyomas from other origins, but share cytogenetic aberrations with non-uterine leiomyomas (Mark et al, 1991). Smooth muscle cells of uterine and non-uterine (blood vessels, gastrointestinal tract, lower urinary tract, vagina) origin share a responsiveness to oestrogen and/or progesterone (Wolf et al, 1991; Everson, 1992; Karas et al, 1994), although there may be differences in expression of receptors for these hormones between smooth muscle cells of various parts of one organ. These features argue for the validity of comparing uterine leiomyomas with leiomyosarcomas from other origins.

In this comparison, it must be concluded that progressing transformation in smooth muscle tumours is associated with complex changes involving gain and loss of expression of various components of the IGF system. Distinct expression patterns of the IGF system are associated with different phenotypes of smooth muscle cell tissues, and therefore it is more likely that transformation to malignant smooth muscle tumours is not a proceeding process, but

© Cancer Research Campaign 1997

that each grade of smooth muscle tumour results from a distinct transformation event. In either case, in this model, the importance of the IGF system for tumorigenesis seems to be restricted to the lower grades of transformation.

### ACKNOWLEDGEMENTS

The authors thank Professor Dr JAM van Unnik for the examination and interpretation of the histological sections. Ruud Bloemen and Ria Reijnen-Gresnigt are acknowledged for their assistance with the immunohistochemistry. This work was supported by the Nederlandse Kankerbestrijding (Dutch Cancer Foundation), Grants UKCC 88-04 and RUU 93-487.

### REFERENCES

Aaronson SA (1991) Growth factors and cancer. *Science* **254**: 1146–1153

Antoniades HN, Galanopoulos T, Neville Golden J and Maxwell M (1992) Expression of insulin-like growth factors I and II and their receptor mRNAs in primary human astrocytomas and meningiomas; in vivo studies using in situ hybridization and immunocytochemistry. *Int J Cancer* **50**: 215–222

Baserga R, Sell C, Porcu P and Rubini M (1994) The role of the IGF-I receptor in the growth and transformation of mammalian cells. *Cell Prolif* **27**: 63–71

Bruning PF, Van Doorn J, Bonfrer JMG, Van Noord PAH, Korse CM, Linders TC and Hart AAM (1995) Insulin-like growth-factor-binding protein 3 is decreased in early-stage operable pre-menopausal breast cancer. *Int J Cancer* **62**: 266–270

Burgess AW (1989) Epidermal growth factor and transforming growth factor alpha. *Br Med Bull* **45**: 401–424

Cohick WS and Clemmons DR (1993) The insulin-like growth factors. *Annu Rev Physiol* **55**: 131–153

Couce ME, Weatherington AJ and McGinty JF (1992) Expression of insulin-like growth factor-II (IGF-II) and IGF-II/mannose-6-phosphate receptor in the rat hippocampus: an in situ hybridization and immunocytochemical study. *Endocrinology* **131**: 1636–1642

Cuthbertson RA, Beck F, Senior PV, Haralambidis J, Penschow JD and Coghlan JP (1989) Insulin-like growth factor II may play a local role in the regulation of ocular size. *Development* **107**: 123–130

D'Ercole AJ and Underwood LE (1987) Estimation of tissue concentrations of Somatomedin C/Insulin-like Growth Factor I. *Methods Enzymol* **146**: 227–233

Denijn M, De Weger RA, Berends MJH, Compier-Spies PI, Jansz HS, Van Unnik JAM and Lips CJM (1990) Detection of calcitonin-encoding mRNA by radioactive and non-radioactive in situ hybridization: improved colorimetric detection and cellular localization of mRNA in thyroid sections. *J Histochem Cytochem* **38**: 351–358

Drop SL, Schuller AG, Lindenbergh Kortleve DJ, Groffen C, Brinkman A and Zwarthoff EC (1992) Structural aspects of the IGFBP family. *Growth Regul* **2**: 69–79

Elliott JL, Oldham JM, Ambler GR, Molan PC, Spencer GSG, Hodgkinson SC, Breier BH, Gluckman PD, Suttie JM and Bass JJ (1993) Receptors for insulin-like growth factor-II in the growing tip of the deer antler. *J Endocrinol* **138**: 233–241

Everson GT (1992) Gastrointestinal motility in pregnancy. *Gastroenterol Clin N Am* **21**: 751–776

Fukuda T, Ohnishi Y, Watanabe H, Kaneko H and Suzuki T (1992) Dedifferentiated leiomyosarcoma of the intestinal tract: histological, ultrastructural and immunohistochemical examinations. *Virchows Arch A* **420**: 313–320

Gelato MC, Kiess W, Lee L, Malozowski S, Rechler MM and Nissley P (1988) The insulin-like growth factor II/mannose-6-phosphate receptor is present in monkey serum. *J Clin Endocrinol Metab* **67**: 669–675

Ghahary A, Chakrabarti S and Murphy LJ (1990) Localization of the sites of synthesis and action of insulin-like growth factor-I in the rat uterus. *Mol Endocrinol* **4**: 191–195

Giudice LC, Irwin JC, Dsupin BA, Pannier EM, Jin IH, Vu TH and Hoffman AR (1993) Insulin-like growth factor (IGF), IGF binding protein (IGFBP), and IGF receptor gene expression and IGFBP synthesis in human uterine leiomyomata. *Hum Reprod* **8**: 1796–1806

Glick RP, Gettleman R, Patel K, Lakshman R and Tsibris JC (1989) Insulin and insulin-like growth factor I in brain tumors: binding and in vitro effects. *Neurosurgery* **24**: 791–797

Gloudemans T, Prinsen I, Van Unnik JA, Lips CJ, Den Otter W and Sussenbach JS (1990) Insulin-like growth factor gene expression in human smooth muscle tumors. *Cancer Res* **50**: 6689–6695

Heldin C-H and Westermark B (1989) Platelet-derived growth factors: a family of isoforms that bind to two distinct receptors. *Br Med Bull* **45**: 453–464

Hsuan JJ (1989) Transforming growth factors beta. *Br Med Bull* **45**: 425–437

Ilvesmaki V, Kahri AI, Miettinen PJ and Voutilainen R (1993) Insulin-like growth factors (IGFs) and their receptors in adrenal tumors: high IGF-II expression in functional adrenocortical carcinomas. *J Clin Endocrinol Metab* **77**: 852–858

Jansen J, Van Buul-Offers SC, Hoogerbrugge CM and Van Den Brande JL (1990) Effects of a single cleavage in insulin-like growth factors I and II on binding to receptors, carrier proteins and antibodies. *Biochem J* **266**: 513–520

Jansen M, Van Schaik FM, Ricker AT, Bullock B, Woods DE, Gabbay KH, Nussbaum AL, Sussenbach JS and Van Den Brande JL (1983) Sequence of cDNA encoding human insulin-like growth factor I precursor. *Nature* **306**: 609–611

Jansen M, Holthuizen P, Van Dijk MA, Van Schaik FMA, Van Den Brande JL and Sussenbach JS (1990) Structure and expression of the insulin-like growth factor II (IGF-II) gene. In *Growth Factors: from Genes to Clinical Application*, Sara VR, Hall K and Löw H (eds), pp. 25–40. Raven Press: New York

Jones JI and Clemmons DR (1995) Insulin-like growth factors and their binding proteins: biological actions. *Endocr Rev* **16**: 3–34

Kaleko M, Rutter WJ and Miller AD (1990) Overexpression of the human insulinlike growth factor I receptor promotes ligand-dependent neoplastic transformation. *Mol Cell Biol* **10**: 464–473

Kapur S, Tamada H, Dey SK and Andrews GK (1992) Expression of insulin-like growth factor-I (IGF-I) and its receptor in the peri-implantation mouse uterus, and cell-specific regulation of IGF-I gene expression by estradiol and progesterone. *Biol Reprod* **46**: 208–219

Karas RH, Patterson BL and Mendelsohn ME (1994) Human vascular smooth muscle cells contain functional estrogen receptor. *Circulation* **89**: 1943–1950

Klempt M, Hutchins AM, Gluckman PD and Skinner SJ (1992) IGF binding protein-2 gene expression and the location of IGF-I and IGF-II in fetal rat lung. *Development* **115**: 765–772

Long L, Rubin R, Baserga R and Brodt P (1995) Loss of the metastatic phenotype in murine carcinoma cells expressing an antisense RNA to the insulin-like growth factor receptor. *Cancer Res* **55**: 1006–1009

Macaulay VM (1992) Insulin-like growth factors and cancer. *Br J Cancer* **65**: 311–320

Mark J, Havel G, Dahlenfors R and Wedell B (1991) Cytogenetics of multiple uterine leiomyomas, parametrial leiomyoma and disseminated peritoneal leiomyomatosis. *Anticancer Res* **11**: 33–39

Merrill MJ and Edwards NA (1990) Insulin-like growth factor-I receptors in human glial tumors. *J Clin Endocrinol Metab* **71**: 199–209

Murphy LJ (1991) The uterine insulin-like growth factor system. In *Modern Concepts of Insulin-Like Growth Factors*, Spencer EM (ed), pp. 275–284. Elsevier Science: New York

Nissley P and Lopaczynski W (1991) Insulin-like growth factor receptors. *Growth Factors* **5**: 29–43

Peyrat JP, Bonneterre J, Vennin PH, Jammes H, Beuscart R, Hecquet B, Djiane J, Lefebvre J and Demaille A (1990) Insulin-like growth factor 1 receptors (IGFI-R) and IGFI in human breast tumors. *J Steroid Biochem Mol Biol* **37**: 823–827

Prager D, Li HL, Asa S and Melmed S (1994) Dominant negative inhibition of tumorigenesis in vivo by human insulin-like growth factor I receptor mutant. *Proc Natl Acad Sci USA* **91**: 2181–2185

Resnicoff M, Coppola D, Sell C, Rubin R, Ferrone S and Baserga R (1994*a*) Growth inhibition of human melanoma cells in nude mice by antisense strategies to the type 1 insulin-like growth factor receptor. *Cancer Res* **54**: 4848–4850

Resnicoff M, Sell C, Rubini M, Coppola D, Ambrose D, Baserga R and Rubin R (1994*b*) Rat glioblastoma cells expressing an antisense RNA to the insulin-like growth factor-1 (IGF-1) receptor are nontumorigenic and induce regression of wild-type tumors. *Cancer Res* **54**: 2218–2222

Rogler CE, Yang D, Rossetti L, Donohoe J, Alt E, Chang CJ, Rosenfeld R, Neely K and Hintz R (1994) Altered body composition and increased frequency of diverse malignancies in insulin-like growth factor-II transgenic mice. *J Biol Chem* **269**: 13779–13784

Roholl PJM, De Jong ASH, Albus-Lutter CE and Van Unnik JAM (1988) Leiomyosarcomas: three cases with desmin positive tumour cells, lacking ultrastructural features of smooth muscle cells. *Histol Histopathol* **3**: 389–394

Roholl PJ, Elbers HR, Prinsen I, Claessens JA and Van Unnik JA (1990) Distribution of actin isoforms in sarcomas: an immunohistochemical study. *Hum Pathol* **21**: 1269–1274

Rosenzweig SA, Zetterstrom C and Benjamin A (1990) Identification of retinal insulin receptors using site-specific antibodies to a carboxyl-terminal peptide of the human insulin receptor alpha-subunit. Up-regulation of neuronal insulin receptors in diabetes. *J Biol Chem* **265**: 18030–18034

Schmandt R and Mills GB (1993) Genomic components of carcinogenesis. *Clin Chem* **39**: 2375–2385

Sell C, Rubini M, Rubin R, Liu JP, Efstratiadis A and Baserga R (1993) Simian virus 40 large tumor antigen is unable to transform mouse embryonic fibroblasts lacking type 1 insulin-like growth factor receptor. *Proc Natl Acad Sci USA* **90**: 11217–11221

Sell C, Dumenil G, Deveaud C, Miura M, Coppola D, Deangelis T, Rubin R, Efstratiadis A and Baserga R (1994) Effect of a null mutation of the insulin-like growth factor I receptor gene on growth and transformation of mouse embryo fibroblasts. *Mol Cell Biol* **14**: 3604–3612

Stevenson KR, Gilmour RS and Wathes DC (1994) Localization of insulin-like growth factor-I (IGF-I) and -II messenger ribonucleic acid and type 1 IGF receptors in the ovine uterus during the estrous cycle and early pregnancy. *Endocrinology* **134**: 1655–1664

Szebenyi G and Rotwein P (1991) Insulin-like growth factors and their receptors in muscle development. *Adv Exp Med Biol* **293**: 289–295

Tommola P, Pekonen F and Rutanen EM (1989) Binding of epidermal growth factor and insulin-like growth factor I in human myometrium and leiomyomata. *Obstet Gynecol* **74**: 658–662

Van Buul-Offers SC, Reijnen-Gresnigt MG, Hoogerbrugge CM, Bloemen RJ, Kuper CF and Van Den Brande JL (1994) Recombinant insulin-like growth factor-II inhibits the growth-stimulating effect of growth hormone on the liver of Snell dwarf mice. *Endocrinology* **135**: 977–985

Van Buul-Offers SC, De Haan K, Reijnen-Gresnigt MG, Meinsma D, Jansen M, Oei SL, Bonte EJ, Sussenbach JS and Van Den Brande JL (1995) Overexpression of human insulin-like growth factor-II in transgenic mice causes increased growth of the thymus. *J Endocrinol* **144**: 491–502

Van Den Brande JL, Hoogerbrugge CM, Beyreuther K, Roepstorff P, Jansen J and Van Buul-Offers SC (1990) Isolation and partial characterization of IGF-like peptides from Cohn fraction IV of human plasma. *Acta Endocrinol* **122**: 683–695

Van Der Ven LTM, Gloudemans T, Roholl PJM, Van Buul-Offers SC, Bladergroen BA, Welters MJP, Sussenbach JS and Den Otter W (1994) Growth advantage of human leiomyoma cells compared to normal smooth muscle cells due to enhanced sensitivity for insulin-like growth factor I. *Int J Cancer* **59**: 427–434

Vollenhoven BJ, Herington AC and Healy DL (1994) Messenger RNA encoding the insulin-like growth factors and their binding proteins, in women with fibroids, pretreated with luteinizing hormone-releasing hormone agonists. *Hum Reprod* **9**: 214–219

Wharton W and Smyth MJ (1989) Growth and maintenance of BALB/c 3T3 cells. In *Cell Growth and Division – A Practical Approach*, Baserga R (ed.), pp. 139–153. IRL Press at Oxford University Press: Oxford

Wilkinson DG and Green J (1992) *In situ* hybridization and the three-dimensional reconstruction of serial sections. In *Postimplantation Mammalian Embryos. A Practical Approach*, Copp AJ and Cockroft DC (eds), pp. 155–171. IRC Press: Oxford

Wolf H, Wandt H and Jonat W (1991) Immunohistochemical evidence of estrogen and progesterone receptors in the female lower urinary tract and comparison with the vagina. *Gynecol Obstet Invest* **32**: 227–231

Yun K (1992) A new marker for rhabdomyosarcoma. Insulin-like growth factor II. *Lab Invest* **67**: 653–664

*© Cancer Research Campaign 1997*